# Tristetraprolin: a weapon against HPV-induced cervical cancer?

**DOI:** 10.18632/aging.100093

**Published:** 2009-10-08

**Authors:** Imed-Eddine Gallouzi, Sergio Di Marco

**Affiliations:** McGill University, Department of Biochemistry, Rosalind and Morris Goodman Cancer Center, Montreal, Quebec, Canada

**Keywords:** cervical cancer, human papillomavirus, mRNA decay, Tristetraprolin, senescence

Cervical cancer, a well-characterized virus-induced malignancy, is the second most common cause of cancer-related death in women [1,2]. While the link between the formation of genital warts and their inherent propensity to progress to cancer was suspected more then a century ago [1], the connection between cervical cancer and the human papillomavirus (HPV) was demonstrated only two decades ago.  During the early 1980's the zur Hausen laboratory provided the first experimental proof that the genome of malignant genital warts was integrated with DNA from the HPV subtypes 16 and 18 [3-5]. In addition to stimulating significant progress in delineating the molecular mechanisms causing cervical cancer, this observation led to the development of an effective vaccine that is now widely used in many countries [2].  However, while this vaccine prevents cervical cancer in ~ 70% of the population [6-10], it is not effective in individuals previously exposed to the virus [2]. Therefore, in order to develop effective strategies to eradicate cervical cancer, additional studies are needed to identify and characterize novel factors and cellular mechanisms involved in HPV-induced malignancy.

It is now well established that to trigger cell transformation, the genome of the high-risk HPV 16 and 18 viruses has to integrate into the DNA of host cells [3-5].  This integration enables the expression of viral proteins such as E6 and E7 which are key players in HPV-induced carcinogenesis as well as in the maintenance of the cell transformation phenotype [11,12].  At the molecular level, E6 and E7 inactivate tumor suppressor mechanisms elicited by p53 and retinoblastoma (pRb) proteins, respectively [2].

Through this influence, E6 and E7 prevent infected cells from entering senescence, a state of irreversible cell cycle arrest that normally occurs at the end of the lifespan of the cell, in the presence of certain oncogenes, or following cellular damage [13]. Additionally, E6 blocks senescence by inducing the expression and activation of the human telomerase reverse transcriptase (hTERT) [14-16]. Although the detailed molecular mechanisms by which E6 promotes malignancy are still elusive, E6 forms a complex with the E6-associated protein (E6-AP), a HECT ubiquitin protein ligase, and mediates the proteasome-dependent degradation of p53. Furthermore, E6-AP has been shown to mediate the degradation of NFX1, a transcriptional repressor of human telomerase reverse transcriptase (hTERT). By decreasing NFX1 levels, E6-AP promotes the expression of hTERT, which consequently increases the activity of the telomerase complex and prevents cellular senescence [2,17]. While links between transcription regulation and the expression of many genes involved in the development of cervical cancer has been described [2,17], the effects of HPV infection on post-transcriptional events such as mRNA turnover are not known.  One of the main questions that remain unanswered to-date is whether infection by HPV negatively or positively affects the mRNA turnover machinery and if so, whether these events are linked to the development of malignant genital warts.

In the September issue ofAGING, Sanduja et al.  [18] answer some of these questions by establishing a direct connection between the ability of the high-risk HPV 18 virus to promote cell malignancy and the inability of the host cell to express the mRNA decay factor tristetraprolin (TTP) [18]. TTP is an RNA-binding protein that binds AU-rich elements (AREs) located in the 3' untranslated region (UTR) of many unstable mRNAs such as those that encode tumor necrosis factor (TNF)-α, granulocyte/macrophage colony-stimulating factor (GM-CSF), IL (interleukin)-2, and cyclooxygenase (Cox)-2 [19].  TTP harbors two cysteine-cysteine-cysteine-histidine zinc-finger domains through which it binds to the AREs of target mRNAs, promoting their rapid degradation via the AMD (ARE-mediated mRNA decay) pathway [20].  Sanduja and colleagues show that TTP levels are substantially low in cervical carcinomas when compared to non-transformed tissue, wherein TTP is highly abundant in the cytoplasm.  These observations argue for a direct link between TTP expression levels and the malignant potential of a cell.  Interestingly, a recent study by the Wilson laboratory showed that TTP expression is low in many cancers, supporting its function as a tumor suppressor, and raising the possibility that TTP abundance could serve as a prognostic indicator of cancer severity and malignancy [21].  On the other hand, Sanduja et al. have observed that expressing TTP protein in cells already infected by HPV leads to cell cycle arrest.  They showed that overexpressing TTP in HeLa (human cervical carcinoma) cells immortalised by the HPV-18 virus causes them to senesce as evidenced by the induction of senescence-associated β-galactosidase (SA β-gal) activity and the significant increase in p53 levels [18].  This effect, however, does not seem to be unique to cells with a potential to develop cervical cancer.  A recent study by the Gorospe laboratory has shown that the expression of TTP is increased in cultured human diploid fibroblasts reaching replicative senescence [22].  As replicative senescence represents a tumor-suppressive mechanism, these findings collectively suggest that preventing the expression of TTP could be an early event in the development of many cancers.  Together with the fact that elevated TTP abundance sensitizes HeLa cells to apoptosis-causing agents such as staurosporine [21], TTP appears to act as a tumor suppressor by promoting senescence and by decreasing the expression of pro-apoptotic proteins.

In an effort to elucidate the molecular mechanism through which TTP induces senescence, Sanduja and colleagues discovered that TTP regulates post-transcriptionally the expression of the E6-AP mRNA [18].  The authors observed that TTP expression in HPV-transformed cells significantly decreased E6-AP mRNA and protein levels.  Although the E6-AP mRNA was quite stable in HeLa cells, it quickly decayed in the presence of TTP.  The ability of TTP to lower the half-life of the E6-AP mRNA was comparable to that observed for other transcripts containing multiple 3'UTR AREs, such as the TNFα mRNA [23,24]. Immunoprecipitation coupled to RT-qPCR experiments confirmed that TTP associates with the E6-AP mRNA in HeLa cells.  Through an elegant set of experiments, the authors demonstrated that TTP regulates post-transcriptionally the E6-AP mRNA via sequences located in its 3'UTR.  Co-expression of TTP with luciferase reporter constructs that either contained or lacked the E6-AP 3'UTR revealed that TTP prevented luciferase expression in the presence of the E6-AP 3'UTR, and that this inhibition was abrogated in the absence of such sequences [18].  Based on this study, it is tempting to conclude that the inhibition of TTP expression is a pre-requisite for the development of HPV-induced cervical cancer.  However, it is also possible that infection by HPV viruses or the expression of oncogenic factors directly reduce TTP levels, resulting in cell transformation. Testing these possibilities experimentally will help to define the exact role of TTP in malignant transformation.

In conclusion, the work by Sanduja et al., and the recent studies described above provide provocative evidence that besides being a potent anti-inflammatory factor [23], TTP could suppress tumor growth by promoting the expression of factors that activate senescence and/or by decreasing the abundance of anti-apoptotic proteins.  The identification of a link between TTP expression and the E6-induced cell transformation provides new insights into the molecular mechanism by which HPV 16 or 18 viruses cause cervical cancer.  In addition, the new evidence points to TTP as a therapeutic target in future strategies to combat HPV-induced malignancies and possibly other cancers.

## Acknowledgements

This work was supported by a CIHR (MOP-67026) and an NCIC (016247) operating grants to I. G. I.G. is a recipient of a TierII Canada Research Chair.

## Conflict of interest statement

The authors of this manuscript have no conflict of interest to declare.
